# Hemodynamics in Coronary Arterial Tree of Serial Stenoses

**DOI:** 10.1371/journal.pone.0163715

**Published:** 2016-09-29

**Authors:** Xi Chen, Yang Gao, Bin Lu, Xinwei Jia, Liang Zhong, Ghassan S. Kassab, Wenchang Tan, Yunlong Huo

**Affiliations:** 1 Department of Mechanics and Engineering Science, College of Engineering, Peking University, Beijing, China; 2 State Key Laboratory for Turbulence and Complex Systems, College of Engineering, Peking University, Beijing, China; 3 State Key Laboratory of Cardiovascular Disease, Fuwai Hospital, National Center for Cardiovascular Diseases, Chinese Academy of Medical Sciences and Peking Union Medical College, Beijing, China; 4 Department of Cardiology, Affiliated hospital of Hebei University, Hebei University, Baoding, China; 5 National Heart Center Singapore, Singapore, Singapore; 6 Duke-NUS Graduate Medical School Singapore, Singapore, Singapore; 7 California Medical Innovations Institute, San Diego, California, United States of America; 8 School of Biomedical Engineering, Capital Medical University, Beijing, China; Universitatsklinikum Wurzburg, GERMANY

## Abstract

Serial segmental narrowing frequently occurs in humans, which alters coronary hemodynamics and further affects atherosclerotic progression and plaque formation. The objective of this study was to understand the distribution of hemodynamic parameters in the epicardial left main coronary arterial (LMCA) tree with serial stenoses reconstructed from patient computer tomography angiography (CTA) images. A finite volume method was used in conjunction with the inlet pressure wave and outlet flow resistance. The time-averaged wall shear stress (TAWSS) and oscillatory shear index (OSI) were determined from the flow field. A stenosis at a mother vessel mainly deteriorated the hemodynamics near the bifurcation while a stenosis at a daughter vessel affected the remote downstream bifurcation. In comparison with a single stenosis, serial stenoses increased the peak pressure gradient along the main trunk of the epicardial left anterior descending arterial tree by > 50%. An increased distance between serial stenoses further increased the peak pressure gradient. These findings have important implications on the diagnosis and treatment of serial coronary stenoses.

## Introduction

Coronary atherosclerosis consisting of diffuse and serial segmental narrowing is frequent in humans [1–5]. The hemodynamic significance of a stenosis can be affected by the presence of other stenoses. In addition to the clinical requirement to understand the individual pressure gradient of each stenosis [3–5], it is also important to understand how serial stenoses interact hemodynamically (e.g., time-averaged wall shear stress-TAWSS and oscillatory shear index-OSI) to affect atherosclerotic progression and potentially plaque rupture.

Low TAWSS and high OSI (i.e., TAWSS < 4 dynes/cm^2^ or OSI > 0.15 [6, 7]) are risk factors for incidence and progression of atherosclerosis [8–10], which can lead to various types of coronary stenoses [11]. Moreover, it has recently been shown that low TAWSS and high OSI are risk factors for rupture-prone phenotype, which may be related to lipid accumulation and inflammatory cell infiltration to the intima [12–14]. Hence, the prediction of hemodynamic changes in coronary arteries (particularly for epicardial arterial trees with complex serial stenoses at coronary bifurcations) is of high significance for understanding atherosclerotic progression and high-risk plaque formation.

Computational fluid dynamics (CFD) simulation has been widely used to predict the distribution of hemodynamic parameters in coronary arteries given the inherent limitation of *in vivo* measurements [15–20]. Recently, the CFD approach has been advocated to determine the fractional flow reserve (FFR) or pressure drop non-invasively [21], which may guide the percutaneous coronary intervention (PCI) for better clinical outcomes [22, 23]. There is, however, lack of studies to determine hemodynamic changes (including the pressure drop) due to serial stenoses of various types at coronary bifurcations.

The objective of this study is to determine the distribution of hemodynamic parameters in patient-specific epicardial left main coronary arterial (LMCA) tree with serial stenoses varying from mild to moderate. The three-dimensional (3D) geometry of patient epicardial LMCA tree was reconstructed from computer tomography angiography (CTA) images. Navier-Stokes and continuity equations were solved using a transient finite volume method. The inlet and outlet boundary conditions were the aortic pressure wave and flow resistance, respectively. Hemodynamic parameters including TAWSS and OSI were determined based on the computed flow field. The significance and limitations of the study were enumerated.

## Materials and Methods

### Study design

**Fig 1:**
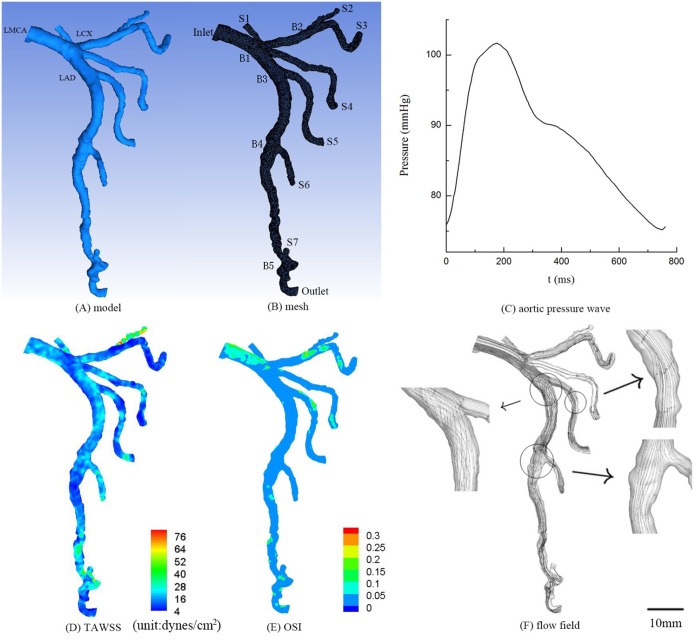
Geometrical model reconstructed from CTA (A), computational meshes (B), measured aortic pressure wave (C), TAWSS (D), OSI (E) and flow field (F) in the epicardial LMCA tree of a healthy subject. The small figures for flow field show the zoomed view.

**Fig 2:**
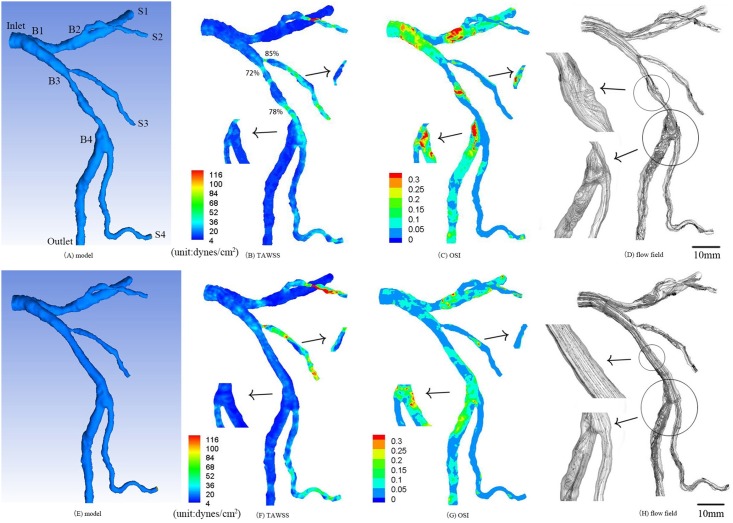
(A-D) Geometrical model reconstructed from CTA (A), TAWSS (B), OSI (C) and flow field (D) in the epicardial LMCA tree of a representative patient who has area stenosis of 72% and stenotic length of 8.1 mm, area stenosis of 78% and stenotic length of 7.9 mm, area stenosis of 85% and stenotic length of 2.4 mm at three sites; (E-H) geometrical model (E), TAWSS (F), OSI (G) and flow field (H) in the epicardial LMCA tree after suppositional angioplasty (i.e., two area stenoses in the main trunk of epicardial LAD tree were assumed to be restored after angioplasty). The small figures for TAWSS and OSI show the posterior view. The small figures for flow field show the zoomed view.

**Fig 3:**
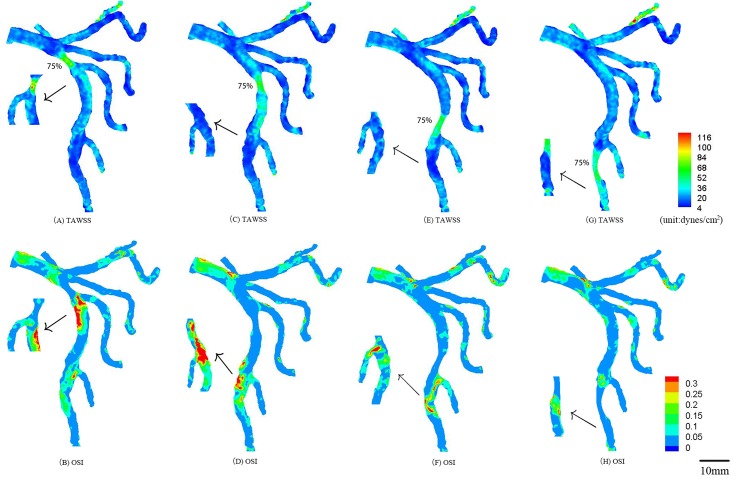
In correspondence with Fig 1A, TAWSS and OSI in the epicardial tree that has an idealized 75% area stenosis at the mother vessel (A-B) and at the large daughter vessel (C-D) (stenotic length of 7.0 and 7.3 mm, respectively) in the first bifurcation of LAD arterial tree; TAWSS and OSI in the epicardial tree that has an idealized 75% area stenosis at the mother vessel (E-F) and at the large daughter vessel (G-H) (stenotic length of 8.5 and 8.9 mm, respectively) in the second bifurcation of LAD arterial tree. The small figures show the posterior view of arteries.

**Fig 4:**
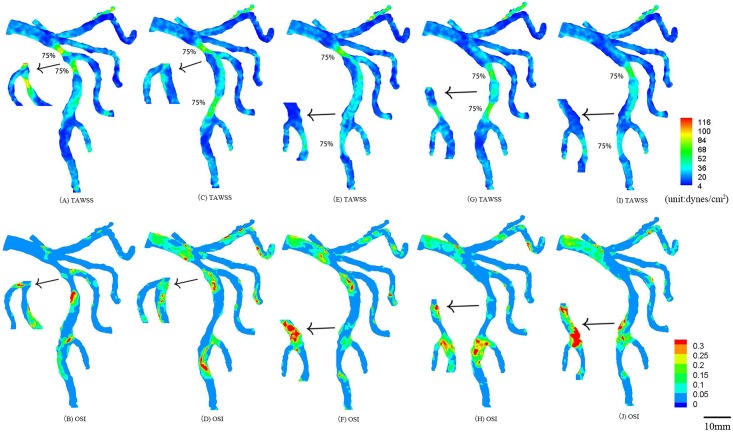
In correspondence with Fig 1A, TAWSS and OSI in the epicardial tree that has two idealized 75% area stenoses at the mother vessel and large daughter vessel (stenotic lengths of 7.0 mm and 7.3 mm) in the first bifurcation of LAD arterial tree (A-B); at the mother vessel in the first and second bifurcations (stenotic lengths of 7.0 mm and 8.5 mm) (C-D); at the mother vessel in the first bifurcation and at the large daughter vessel in the second bifurcation (stenotic lengths of 7.0 mm and 8.9 mm) (E-F); at the large daughter vessel in the first bifurcation and at the mother vessel in the second bifurcation (stenotic lengths of 7.3 mm and 8.5 mm) (G-H); at the large daughter vessel in the first and second bifurcations (stenotic lengths of 7.3 mm and 8.9 mm) (I-J). The small figures show the posterior view of arteries.

**Fig 5:**
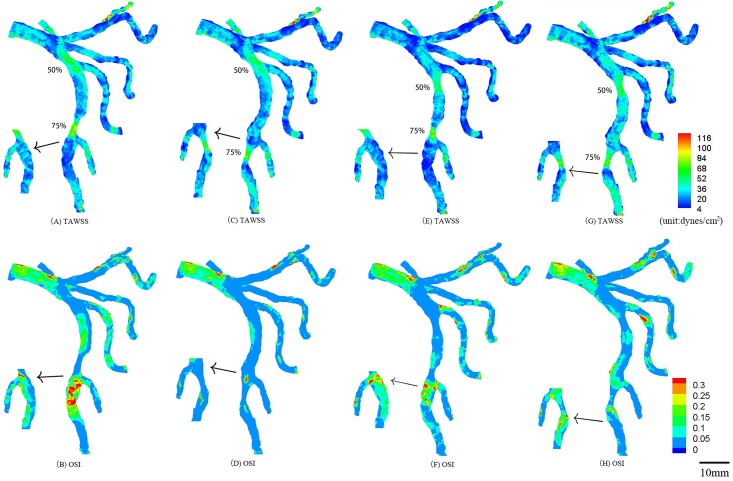
In correspondence with Fig 1A, TAWSS and OSI in the epicardial tree that has an idealized 50% area stenosis of 7.0 mm length at the mother vessel of the first bifurcation and an idealized 75% area stenosis of 8.5 mm length at the mother vessel of the second bifurcation in the LAD arterial tree (A-B); an idealized 50% area stenosis of 7.0 mm length at the mother vessel of the first bifurcation and an idealized 75% area stenosis of 8.9 mm length at the large daughter vessel of the second bifurcation in the LAD arterial tree (C-D); an idealized 50% area stenosis of 7.3 mm length at the large daughter vessel of the first bifurcation and an idealized 75% area stenosis of 8.5 mm length at the mother vessel of the second bifurcation in the LAD arterial tree (E-F); an idealized 50% area stenosis of 7.3 mm length at the large daughter vessel of the first bifurcation and an idealized 75% area stenosis of 8.9 mm length at the large daughter vessel of the second bifurcation in the LAD arterial tree (G-H). The small figures show the posterior view of arteries.

**Fig 6:**
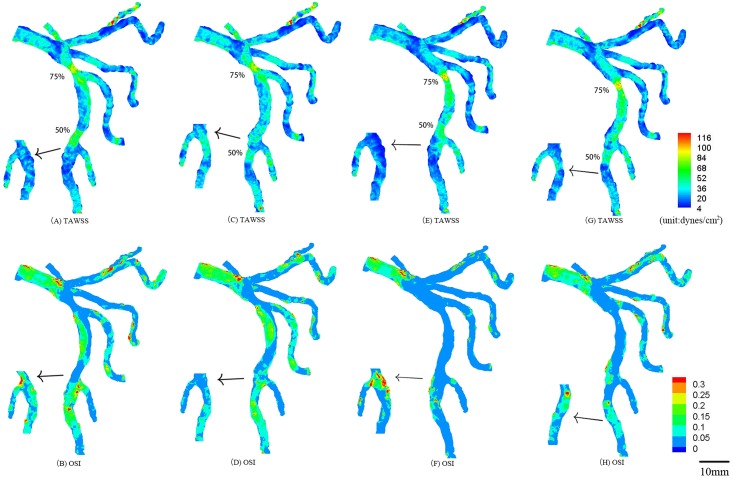
TAWSS and OSI in the epicardial tree that has an idealized 75% area stenosis at the first bifurcation and an idealized 50% area stenosis at the second bifurcation in the LAD arterial tree corresponding to Fig 5A–5H. The small figures show the posterior view of arteries.

The aim of this retrospective study was to investigate hemodynamic changes in serial coronary stenoses. Two human subjects, as shown in Figs 1A and 2A, underwent the CTA of coronary arteries. The retrospective study was approved by the Institutional Review Board (IRB) for the cardiovascular Institute and Fu Wai Hospital, Chinese Academy of Medical Sciences, Peking Union Medical College. Human subjects gave the signed informed consent. Furthermore, two types of idealized stenoses (i.e., 50% and 75% area stenoses as the most typical stenoses in patients) were created in the healthy epicardial LAD (left anterior descending) arterial tree in Fig 1A to investigate the effects of serial stenoses on hemodynamics, as shown in Figs 3–6. Serial stenoses were also removed from the diseased epicardial LAD arterial tree in Fig 2A to mimic the restoration after angioplasty, as shown in Fig 2E–2H.

### Imaging acquisition

Similar to a previous study [24], prior to imaging acquisition, patients were given the repeated doses of intravenous metoprolol of 5 mg every 5 minutes until heart rate was ≤ 65 bpm or a maximum dose of 15 mg was given. All patients received sublingual nitroglycerin tablet (0.4 mg) 3–5 minutes before CTA examination.

All studies were performed on a dual-source CT scanner (Siemens Definition, Forchheim Germany). After an initial survey scan, a retrospectively gated contrast-enhanced scan was obtained using 80 ml of iodinated contrast (Iopromide-Ultravist 370, Bayer Healthcare, Morristown USA) injected through an antecubital vein at 5 ml/s followed by 50 ml of normal saline at the same rate. The scan parameters were: 2 × 64 × 0.6 mm collimation, tube voltage– 120 kV; tube current–average 620 mAs adjusted to body size; gantry rotation time– 330 msec; pitch– 0.2–0.43 depending on heart rate. The simultaneous acquisition of multi-parallel cross sections enabled the imaging of coronary artery in a single breath hold. Images were reconstructed with a slice thickness/increment of 0.7/0.4 mm with B26f at temporal resolution of 83 msec (half-scan). The initial data window was positioned at 70% of the R-R interval, with additional data sets reconstructed at ±5% intervals to compensate for motion artifacts in coronary arteries if necessary.

### Geometrical models

Morphometry of the epicardial LMCA tree was extracted from patients CTA images using the MIMICS software (Materialise, NV, Belgium). In the MIMICS software, a centerline was formed by a series of center points which was located in the center on the cross–sectional views of the contour of the 3D vessel. Subsequently, the best fit diameter, D_fit_, was calculated as twice the average radius between the point on the centerline and the contour forming the 3D vessel.

Geomagic Studio software (3D Systems, Rock Hill, USA) was used to generate geometrical models that were meshed using ANSYS ICEM (ANSYS, Canonsburg, USA), as shown in Fig 1B. A mesh dependency was conducted such that the relative error in two consecutive mesh refinements was < 1% for the maximum velocity of steady state flow with inlet flow velocity equal to the time-averaged velocity over a cardiac cycle. A total of approximately 500,000 tetrahedral shaped volume elements (element size = 0.2 mm) were necessary to accurately mesh the computational domain.

### 3-D computational model

Similar to a previous study [9], governing equations were formulated for coronary arteries, each vessel of which was assumed to be rigid and impermeable. Navier-Stokes and continuity equations were solved using the commercial software solver FLUENT (ANSYS, Inc., Canonsburg, USA). Three cardiac cycles were required to achieve convergence for the transient analysis similar to previous studies [9, 17]. The implicit Euler method was used and a constant time step was employed, where Δt = 0.01 s with 80 total time step per cardiac cycle. Although blood is a suspension of particles, it behaves as a Newtonian fluid in vessels with diameters > 1 mm [19]. The measured aortic pressure wave in Fig 1C was set as the boundary condition at the inlet of LMCA trees. The resistance boundary condition was assigned to each outlet (see the Appendix of Ref. [9]). The viscosity and density were assumed as 4.5×10^−3^ Pa·s and 1060 kg/m^3^, respectively, to mimic blood flow with a hematocrit of about 45% in these arteries. After the velocity and pressure of blood flow were calculated, hemodynamic parameters including TAWSS and OSI were determined from the equations in the Appendix of Ref. [9]. Moreover, SAR-TAWSS (i.e., surface area ratio of TAWSS that equals to $\frac{{\text{Surface area}}_{\text{TAWSS}\leq \text{4 dynes}\cdot {\text{cm}}^{\text{-2}}}}{\text{Surface area near a bifurcation}}\;\times 100\%$) and SAR-OSI (i.e., surface area ratio of high OSI that equals to $\frac{{\text{Surface area}}_{\text{OSI}\geq \text{0}.15}}{\text{Surface area near a bifurcation}}\times 100\%$) were computed at coronary bifurcations using the method in Ref. [9]. The peak pressure gradient (i.e., the pressure gradient at the peak flow rate) was computed through each stenosis.

## Results

CFD simulations were performed in the reconstructed epicardial LMCA trees of two human subjects, as shown in Figs 1A and 2A. Fig 1D–1F show the distribution of TAWSS, OSI and flow field, respectively, in the epicardial LMCA tree of a healthy subject. Fig 2B–2D show those hemodynamic parameters in a patient with serial stenoses, which lead to an increased pressure drop as well as increased OSI and decreased WSS downstream of the stenoses. The corresponding morphometric and hemodynamic parameters are listed in Table 1. Moreover, the two stenoses in the main trunk of LAD tree in Fig 2A were removed to mimic the restoration after angioplasty, which altered the distribution of TAWSS, OSI and flow field, as shown in Fig 2E–2H.

**Table 1:** Length of the main trunk and primary branches, diameter and mean velocity (time-averaged velocity over a cardiac cycle) at the inlet and each outlet in the epicardial LMCA tree of patients in Figs 1 and 2.

	Vessel Length (mm)			Diameter (mm)	Velocity(cm/s)
**Patient in** Fig 1			Inlet	4.325	3.652
	B1-S1	6.59	S1	1.481	2.265
	B2-S2	10.81	S2	1.212	2.646
	B2-S3	26.51	S3	1.405	2.553
	B1-S4	36.42	S4	2.102	2.183
	B3-S5	30.54	S5	1.924	2.181
	B4-S6	18.14	S6	1.668	2.432
	B5-S7	9.32	S7	1.596	2.509
	Inlet-Outlet	105.84	Outlet	3.191	2.296
**Patient in** Fig 2			Inlet	5.137	3.634
	B2-S1	19.23	S1	3.548	3.052
	B2-S2	22.95	S2	1.789	3.337
	B3-S3	38.14	S3	1.211	3.346
	B4-S4	53.75	S4	1.801	3.279
	Inlet-Outlet	88.05	Outlet	3.085	3.252

**Inlet**: the most proximal position of the reconstructed epicardial LMCA tree, as shown in Figs 1B and 2A

**Outlet**: the most distal position of the main trunk of the reconstructed epicardial LAD tree, as shown in Figs 1B and 2A

**S1-S7**: the outlets of primary branches of the epicardial LMCA tree, as shown in Figs 1B and 2A

**B1-B5**: the inlets of primary branches of the epicardial LMCA tree, as shown in Figs 1B and 2A

TAWSS and OSI were computed in the epicardial LMCA tree that had an idealized 75% area stenosis at the mother vessel (or the large daughter vessel) in the first or second LAD bifurcation, as shown in Fig 3A–3H. The two parameters were also computed in the epicardial LMCA tree that had serial stenoses (50% and 75% area stenoses) at the mother vessel (or the large daughter vessel) in the first and second LAD bifurcations, as shown in Figs 4–6. Accordingly, Table 2 lists the peak pressure gradient along the epicardial LAD main trunk (i.e., aortic peak pressure—outlet peak pressure, where ‘outlet’ refers to the most distal position along the epicardial LAD main trunk). In comparison with a single stenosis, serial stenoses in the LAD main trunk increased the pressure drop by > 50% while a decreased distance between serial stenoses reduced the pressure drop, as shown in Table 2.

**Table 2:** Peak pressure gradient along the epicardial LAD main trunk (i.e., aortic peak pressure—outlet peak pressure).

	Peak pressure gradient (Pa)				
**75% in** Fig 3	Case 1	Case 2	Case 3	Case 4	
	681	688	720	735	
**75%+75% in** Fig 4	Case 5	Case 6	Case 7	Case 8	Case 9
	1148	1202	1235	1288	1295
**50%+75% in** Fig 5	Case 10	Case 11	Case 12	Case 13	
	974	988	1035	1042	
**75%+50% in** Fig 6	Case 14	Case 15	Case 16	Case 17	
	938	950	985	990	

Case 1: a stenosis in Fig 3A;

Case 2: a stenosis in Fig 3C;

Case 3: a stenosis in Fig 3E;

Case 4: a stenosis in Fig 3G;

Case 5: serial stenoses in Fig 4A;

Case 6: serial stenoses in Fig 4G;

Case 7: serial stenoses in Fig 4I;

Case 8: serial stenoses in Fig 4C;

Case 9: serial stenoses in Fig 4E

Case 10: serial stenoses in Fig 5E;

Case 11: serial stenoses in Fig 5G;

Case 12: serial stenoses in Fig 5A;

Case 13: serial stenoses in Fig 5C;

Case 14: serial stenoses in Fig 6E;

Case 15: serial stenoses in Fig 6G;

Case 16: serial stenoses in Fig 6A;

Case 17: serial stenoses in Fig 6C;

## Discussion

This study performed CFD simulations in patient epicardial LMCA tree, based on which the hemodynamic analysis was carried out in coronary bifurcations of stenoses along the LAD main trunk given most stenoses occurring near bifurcations (or trifurcations) [11, 25]. As shown in Fig 2D, the stenoses led to complex flow patterns (significantly increased flow vortices and secondary flows distal to the stenoses) and deteriorated hemodynamic conditions (i.e., decreased TAWSS and increased OSI) and enlarged atherosclerosis-prone zones (i.e., an increase of SAR-TAWSS or SAR-OSI) in the epicardial LAD arterial tree. In comparison with Fig 1, the stenoses in Fig 2 resulted in an approximately 4-fold increase of SAR-TAWSS as well as 20–50% SAR-OSI in coronary bifurcations along the LAD main trunk in contrast with ~0% SAR-OSI in normal case. Furthermore, the simulated angioplasty could only restore the hemodynamics to a certain extent because there were still strong secondary flows in the second LAD bifurcation (Figs 2H vs. 1F), which may be a risk factor for restenosis after PCI and needs further investigations.

The hemodynamic analysis was carried out in an idealized stenosis with 75% area stenosis in the first or second bifurcation of the LAD arterial tree, as shown in Fig 3. The stenosis at the mother vessel extended the atherosclerosis-prone zones to the entire bifurcation. The stenosis at the daughter vessel significantly decreased TAWSS and increased OSI at the remote downstream bifurcation, e.g., the stenosis at the large daughter vessel of the first LAD bifurcation deteriorated the hemodynamics at the second bifurcation. The peak pressure gradients along the epicardial LAD main trunk were similar to each other for mother and daughter stenoses (Cases 1 vs. 2 or Cases 3 vs. 4 in Table 2). Moreover, the 75% area stenosis in the second LAD bifurcation resulted in the higher pressure gradient as compared with that in the first bifurcation because of the relatively large lumen size in the first bifurcation, which agreed with the theoretical prediction [26].

Serial moderate stenoses at mother and daughter vessels of the first LAD bifurcation in Fig 4A partially improved the hemodynamic conditions near the bifurcation (e.g., ~50% reduction of SAR-TAWSS and SAR-OSI) as compared with a single stenosis, which was consistent with previous studies [19, 27]. The reduced lumen CSA (cross-sectional area) increased the flow velocity and led to a larger inertial force and thus increased TAWSS and decreased OSI inside the stenosis significantly, which resulted in the decrease of SAR-TAWSS and SAR-OSI near the bifurcation. The serial stenoses also increased the peak pressure gradient by > 50% as compared with the single stenosis (Case 5 vs. Cases 1–4 in Table 2).

Serial stenoses at mother vessels of the first and second bifurcations decreased TAWSS, increased OSI, and extended atherosclerosis-prone zones near its own bifurcation in the LAD arterial tree, but had relatively small effects on each other, as shown in Figs 4C, 5A and 6A. Similarly, serial stenoses at daughter vessels of the first and second LAD bifurcations had relatively small effects on each other, as shown in Figs 4I, 5G and 6G. There was negligible interaction between serial stenoses at the mother vessel in the first bifurcation and at the daughter vessel in the second bifurcation, as shown in Figs 4E, 5C and 6C. Serial stenoses at the daughter vessel in the first bifurcation and at the mother vessel in the second bifurcation showed strong interaction similar to serial stenoses at a bifurcation. On the other hand, the increased distance between serial stenoses along the LAD main trunk reduced the interaction between them and resulted in the increase of peak pressure gradient, i.e., from case 5 to case 9, from case 10 to case 13 and from case 14 to case 17 in Table 2, which agreed with clinical observations [28].

### Implications for clinical applications

A key finding of the study was to show the increased hemodynamic interaction of serial stenoses along the main trunk of LAD as the distance between them was reduced. Interventional cardiologist requires to consider the interaction for the diagnosis and treatment of serial coronary stenoses. For example, the fractional flow reserve (i.e., $\text{FFR}=\frac{\text{Presse distal to a coronary stenosis}}{\text{Aortic pressure}}$) has been proposed to assess the hemodynamic significance of a coronary stenosis [22, 23]. For serial stenoses in Figs 4G, 5E and 6E, after a primary stenosis was treated, the hemodynamic environment of an untreated stenosis was altered such that the corresponding FFR should be reassessed. For serial stenoses in Figs 5C and 6C, a single pullback pressure recording determined the FFR through each stenosis given the negligible interaction between them.

Another key finding was that serial moderate stenoses at mother and daughter vessels of a coronary bifurcation enhanced the hemodynamic conditions near the bifurcation as compared with a single stenosis. The two key findings suggest that a long stent placement should be selected to cover the serial stenoses in Fig 4A when FFR < 0.75, which can reduce the surface area with disturbed flows to retard the progression of atherosclerotic plaques as well as avoid the unnecessary reassessment of FFR during the PCI procedure.

### Critique of model

A previous study has shown that vessel compliance has relatively small effects on TAWSS and OSI in normal coronary arteries [17]. The stenosis, however, reduces the vessel compliance as compared with normal coronary vessels [29]. The compliance mismatch between stenotic and normal vessels should be incorporated in future numerical simulation studies, which can potentially enhance the stent selection (e.g., drug-eluting stents [30] or bio-absorbable stents [31]) to reduce modality and mortality after percutaneous coronary intervention. Furthermore, future validations of the computational results are needed by measurements of coronary pressure and flow velocities [28, 32].

## Conclusions

Serial moderate stenoses along the LAD main trunk increased the peak pressure gradient by > 50% in comparison with a single stenosis, where the increased distance between serial stenoses increased the peak pressure gradient. Moreover, a stenosis at a mother vessel was found to mainly worsen the hemodynamic distribution near the bifurcation while a stenosis at a daughter vessel affected the remote downstream bifurcation. For serial stenoses, the downstream stenosis enhanced the hemodynamics impaired by the upstream stenosis, but the proximal and distal sites to the downstream stenosis had lower TAWSS and higher OSI. The hemodynamic analysis in multiple stenoses of the epicardial coronary arterial tree improves our understandings of diffuse atherosclerotic progression.
